# Different Modes of Mechanism of Gamma-Mangostin and Alpha-Mangostin to Inhibit Cell Migration of Triple-Negative Breast Cancer Cells Concerning *CXCR4 *Downregulation and ROS Generation

**DOI:** 10.5812/ijpr-138856

**Published:** 2023-11-10

**Authors:** Sarmoko Sarmoko, Dhania Novitasari, Manami Toriyama, Muhamad Salman Fareza, Nur Amalia Choironi, Hiroshi Itoh, Edy Meiyanto

**Affiliations:** 1 Department of Pharmacy, Sumatera Institute of Technology, Lampung, Indonesia; 2 Cancer Chemoprevention Research Center, Faculty of Pharmacy, Universitas Gadjah Mada, Indonesia; 3 Laboratory of Tumor Cell Biology, Division of Biological Science, Graduate School of Science and Technology, Nara Institute of Science and Technology, Japan; 4 Laboratory of Molecular Signal Transduction, Nara Institute of Science and Technology, Japan; 5 Laboratory of Advanced Cosmetic Science, Graduate School of Pharmaceutical Science, Osaka University, Japan; 6 Department of Pharmacy, Jenderal Soedirman University, Indonesia; 7 Department of Pharmaceutical Chemistry, Faculty of Pharmacy, Universitas Gadjah Mada, Indonesia

**Keywords:** Garcinia Mangostana, Cell Migration Inhibition, Breast Neoplasm, Computational Biology

## Abstract

**Background:**

Two mangostin compounds, gamma-mangostin and alpha-mangostin, show anticancer properties through the inhibition of cell proliferation and cell migration. Metastatic triple-negative breast cancer (TNBC) cells, including MDA-MB-231, highly express C-X-C chemokine receptor type 4 (CXCR4) to maintain reactive oxygen species (ROS) and cell migration.

**Objectives:**

This study was performed to analyze and compare different modes of action of γ-mangostin and α-mangostin as antimigratory effects targeted on *CXCR4 *in MDA-MB-231 as a model of TNBC cell.

**Methods:**

This study investigated the effect of γ-mangostin and α-mangostin using a series of assays, including Cell Counting Kit-8 (CCK-8) assay for cytotoxicity, wound healing assay for migration study, quantitative real-time polymerase chain reaction (qRT-PCR) for gene expression analysis, and flow cytometry for ROS measurement, along with in silico study to observe the binding between the compound and CXCR4.

**Results:**

The findings revealed half maximal inhibitory concentration (IC50) values of 25 and 20 μM for γ-mangostin and α-mangostin in MDA-MB 231 cells, respectively. Moreover, a concentration of 10 μM was used for the migration assay. Both γ-mangostin and α-mangostin significantly suppressed cell migration within 24 hours. The present gene expression studies revealed the downregulation of key migration-associated genes, namely *Farp*, CXCR4, and *LPHN2*, upon γ-mangostin treatment but not α-mangostin. Additionally, both γ-mangostin and α-mangostin increased cellular ROS generation, highlighting the same effect of γ-mangostin and α-mangostin ROS elevation to inhibit cancer cell migration. Molecular docking simulations further suggested a potential interaction between γ-mangostin and α-mangostin with *CXCR4 *in high affinity.

**Conclusions:**

These findings suggest that both γ-mangostin and α-mangostin inhibit breast cancer cell migration and induce cellular ROS levels in MDA-MB-231 cells; notably, γ-mangostin suppresses *CXCR4 *mRNA expression that might correlate to its activity to inhibit MDA-MB-231 cell migration.

## 1. Background

The metastatic process in breast cancer involves complex interactions regulated by various gene expressions (1) and is closely associated with cancer cell migration and invasion. Triple-negative breast cancer (TNBC), a particularly invasive subtype, constitutes 15 - 20% of total diagnosed cases (2). Typically, TNBC has a poor prognosis due to a lack of targeted therapies to cure this subtype. Standard treatment for most TNBC cases involves broad-spectrum cytotoxic chemotherapy drugs, surgery, and radiotherapy (3, 4); however, more than 60% of TNBC patients do not respond to these therapies (5, 6). Clinically, TNBC is commonly treated with chemotherapy drugs, such as anthracyclines, taxanes, capecitabine, or eribulin; nevertheless, these drugs often cause side effects and aggravate patients’ conditions due to their non-specific action on cancer cells (7, 8). Pembrolizumab, a novel immunotherapy approved for early-stage TNBC, is frequently associated with inflammation-related side effects (9).

Recent studies conclude that the TNBC subtype exhibits elevated reactive oxygen species (ROS) due to mitochondrial dysfunction, contributing to their high malignancy through impacts on the tumor microenvironment and cancer cell survival (10, 11). Interestingly, targeting oxidative cellular stress can promote cancer cell death, as observed with chemotherapeutics that induce ROS overproduction and activate the apoptotic pathway (12). Therefore, developing novel drugs that specifically target or interfere with cancer cell metabolism, leading to cancer cell death with minimal side effects and a low risk of relapse, could be beneficial in effectively treating TNBC.

One such target is CXCR4, a gene encoding the C-X-C chemokine receptor type 4 (CXCR4) protein, which plays an essential role in the metastasis of cancer cells. The *CXCR4 *expression is significantly increased in many types of malignant cancers, including TNBC (13, 14). The CXCR4 protein, part of the G-protein coupled receptors (GPCR) family, forms complexes with CXCL12 (a chemokine as its ligand) and transmits signals to activate several genes involved in cell migration and invasion, such as matrix metalloproteinases (MMPs) (15, 16). The *CXCR4 *also plays a role in the accumulation and regulation of ROS levels in cancer cells, protecting cells against cellular damage from oxidative stress (17). Therefore, *CXCR4 *represents an attractive target for developing metastatic anticancer agents.

Several agents targeting *CXCR4 *have been developed, such as BPRCX807, a synthetic compound prepared from AMD1300 modification. Plerixafor (AMD3100), a United States Food and Drug Administration (FDA)-approved *CXCR4 *antagonist (18), is not an anticancer drug and has limitations, such as toxicity and poor pharmacokinetics. BPRCX807 can effectively interact with *CXCR4 *and inhibit its activity more efficiently than plerixafor, thereby helping prevent the proliferation and migration of hepatocellular carcinoma cells. However, BPRCX807 has a complex structure, making its synthesis challenging (19). Therefore, there remains a prospect to develop other compounds that are easier to provide, with natural products offering promising potential.

The present study explored active natural compounds from mangostin fruit (*Garcinia mangostana* L.), which have been widely studied for their antitumor activities (20). The fruit pericarp of mangostin contains alph-mangostin, beta-mangostin, and gamma-mangostin, which show promise for anticancer. Alpha-mangostin, in particular, has been extensively studied for its activity against breast cancer (21). This compound inhibits the invasion and metastasis of TNBC cells (22) and downregulates MMPs, including MMP-2 and MMP-9 (23). Moreover, α-mangostin targets multiple signaling pathways involved in cell cycle regulation, proliferation, and metastasis inhibition (24). However, the specific targets of α-mangostin in suppressing cancer cell proliferation and migration remain unknown. Gamma-mangostin, on the other hand, has been less explored despite its demonstrated effectiveness against colon cancer (25). Flow cytometry-based analysis revealed an increase in hypodiploid cells in γ-mangostin-treated HT29 colorectal adenocarcinoma cells, suggesting apoptosis induction (25). Given the minor structural differences between γ-mangostin and α-mangostin, this study hypothesized that these compounds might have a similar effect on inhibiting breast cancer cell migration, particularly regarding ROS level and *CXCR4 *expression. The MDA-MB-231 cell line was used in this study as a model of TNBC to explore the potential of these natural compounds as specific-target agents for metastatic breast cancer.

## 2. Objectives

The present study aimed to investigate the differential mechanism of γ-mangostin and α-mangostin as potential agents against metastatic MDA-MB-231 breast cancer cells and focus on cancer cell migration and its modulation to cellular ROS, followed by determining its effect on expression in cell migration-associated genes, including CXCR4. Using the insights provided by the emergent field of bioinformatics, molecular docking was also conducted to analyze the interaction of γ-mangostin and α-mangostin with the protein target.

## 3. Methods

### 3.1. Cell Cultures

The MDA-MB-231 breast cancer cells were cultured in an L-15 medium, enriched with 15% fetal bovine serum (FBS) (Gibco, USA) and 1% penicillin-streptomycin (Gibco, USA), and incubated in CO_2_-free 37°C incubator.

### 3.2. Cell Viability Assay

The compounds γ-mangostin (#M6824) and α-mangostin (#M3824) were purchased from Sigma-Aldrich (USA). A suspension of cells (20,000 cells/well in 100 μL) was cultured in a 96-well plate. After an initial 24-hour incubation, γ-mangostin or α-mangostin of varying concentrations (1 - 100 μM) was added to the plate, followed by another 24-hour incubation. A solution of Cell Counting Kit-8 (CCK-8) (Dojindo #347-07621, Japan) was added to each well, and the plate was incubated for 2 hours. The absorbance was subsequently read at 450 nm using a microplate reader (26). The assay was carried out in triplicate.

### 3.3. Migration Assay

A culture-insert 2 well (cat #ib81176) was placed in the well. Then, 5 × 10^4^ cells were seeded on each side of the insert. After 24 hours, the medium was discarded and replaced with the fresh medium containing 0.5% FBS, and the cells were cultured for another 18 hours. The next day, the medium was replaced with 10 µg/mL mitomycin C (Wako #139-18711, Japan) and cultured for 2 hours before treatment with 10 μM γ-mangostin and 10 μM α-mangostin. Right after the treatment, the cells were observed under the microscope at 0 hour. At the indicated times (24 and 42 hours), the cell-free area from three parts (i.e., upper, center, and lower scratch) was measured by analyzing the gap closure rate using ImageJ software (27).

### 3.4. Quantitative Polymerase Chain Reaction

Total ribonucleic acid (RNA) upon treatment with γ-mangostin and α-mangostin was collected from cells using Sepasol (Nacalai Tesque, Japan). The reverse transcription of complementary deoxyribonucleic acid (cDNA) was performed using ReverTra Ace qPCR RT Kit (Toyobo, Japan), which included DNAse I to remove genomic DNA. Quantitative real-time reverse-transcription polymerase chain reaction (qRT-PCR) was performed using the Thunderbird SYBR qPCR Mix (Toyobo, Japan) on a Real-Time PCR System Light Cycler 96 (Roche, USA). Rac, *Farp*, *CXCR4 *(28), and latrophilin-2 (*LPHN2*) primers were used, and *GAPDH* was detected as the internal control. The primer sequences are listed in Table 1. 

**Table 1.** Primer Sequences ^a^

Genes and Primer Sequences	NCBI Reference Sequence	The Size (bp) of the Expected Amplicon	TM°C
***hRac1***			
Fw: 5’-CCTTGTGAGTCCTGCATCATTTG-3’	NM_006908.5	149	60.10
Rv: 5’-TCTTCTCCTTCAGTTTCTCGATCG-3’			60.14
***h** **Farp***			
Fw: 5’-CCCAGGAGGCATTTGAAGTTCC-3’	NM_005766.4	99	61.47
Rv: 5’-GGCCAAAATAGTCACCTTCCACG-3’			61.97
***hCXCR4***			
Fw: 5’-CTGCGAGCAGAGGGTCCAG-3’	XM_047445802.1	60	62.37
Rv: 5’-ATGAATGTCCACCTCGCTT-3’			56.73
***h** **LPHN2***			
Fw: 5’-TGATGCTGACCCATTTCAGA-3’	XM_054335486.1	138	56.82
Rv: 5’-CCAGGACATGGATCAGGAAA-3’			56.89
***h** **GAPDH***			
Fw: 5’-GGCTGAGAACGGGAAGCTTG-3’	NM_001357943.2	110	61.38
Rv: 5’-ACTCCACGACGTACTCAGCG-3’			61.91

^a^ NCBI, National Center for Biotechnology Information.

### 3.5. Intracellular ROS Level Measurement

The MDA-MB-231 cells were collected in a buffer with 10% FBS in phosphate-buffered saline (PBS) and transferred in a sterile light-blocking microtube before being stained with 20 µM of 2’,7’-dichlorofluorescein diacetate (DCFDA) (Sigma #D6883, USA) and incubated for 30 minutes. Later, γ-mangostin and α-mangostin were added to the cell suspensions and incubated in a 37ºC incubator (with 5% CO_2_) before analyzing by a flow cytometer (Calibur) at indicated times (4, 8, 18, and 24 hours). 2’,7’-dichlorofluorescein diacetate reacted with the hydroxyl radicals (·OH) and transformed into fluorescent 2,7-dichlorofluorescein (DCF). The fluorescence intensity of DCF based on 5,000 cells per gate was calculated using the flow cytometer (29, 30). In a separate experiment, the cells already stained with DCFDA were treated with 5 mM N-acetyl-L-cysteine (NAC) (Wako #017-05131, Japan) and incubated for an hour before the treatment with γ-mangostin and α-mangostin for the next 24 hours, and the ROS level was subsequently measured. The assay was carried out in triplicate.

### 3.6. Molecular Docking

Molecular Operating Environment (MOE) software (version 2010.12) was employed to predict the molecular interaction between γ-mangostin or α-mangostin with *CXCR4 *(PDB ID: 3ODU). Gamma-mangostin and α-mangostin were prepared into a single dataset and selected a partial charge by MMFF94X force field parameters. The docking protocol used was based on a study by Meiyanto et al. (31), with the ligand conformation having the lowest docking score selected for binding interaction analysis.

### 3.7. Data Analysis

The data were presented as graphs using GraphPad Prism software (version 9.0) and were statistically analyzed using one-way analysis of variance (ANOVA) followed by the Tukey post hoc test. A confidence level of P < 0.05 was considered significant.

## 4. Results

### 4.1. Cytotoxic Effect of Gamma-Mangostin and Alpha-Mangostin on MDA-MB-231 Cells

**Figure 1.**
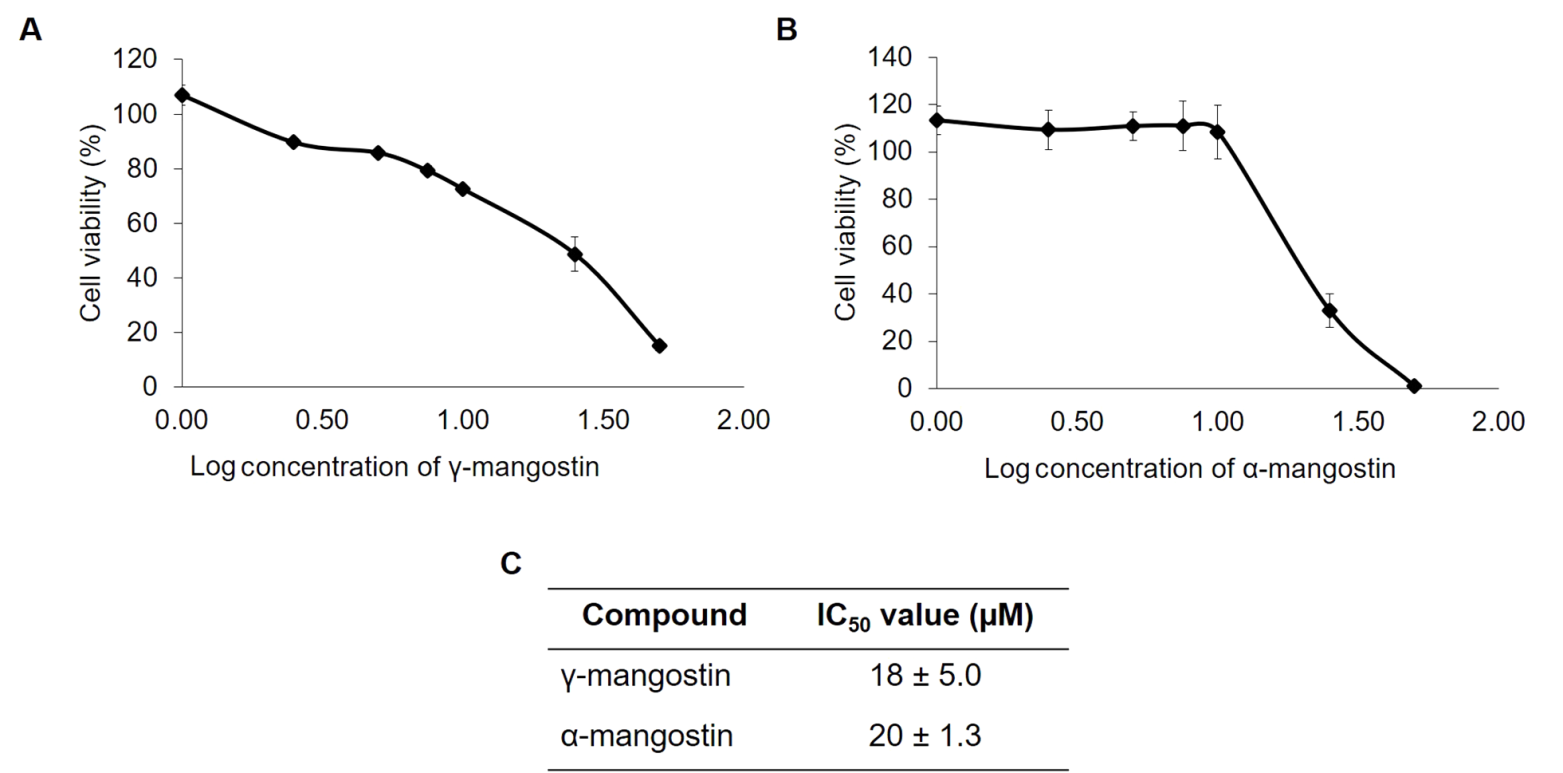
Cytotoxic effect of gamma-mangostin and alpha-mangostin on MDA-MB-231 cells; A, gamma-mangostin and B, alpha-mangostin treatment for MDA-MB-231 cells for 24 hours. Later, the cell viability was assessed using CCK-8 assay. The absorbance was converted into the percent of cell viability and calculated for the IC50 value. The data are presented as the average of triplicate ± standard deviation (SD).

First, the toxicity of γ-mangostin and α-mangostin were analyzed using the CCK-8 assay in MDA-MB 231 cells. The results indicated that γ-mangostin and α-mangostin suppressed MDA-MB-231 cell proliferation (Figure 1A and B), giving the half maximal inhibitory concentration (IC_50_) values of 18 ± 5.0 and 20 ± 1.3 μM, respectively (Figure 1C). It was realized that treating cells with concentrations higher than 25 μM (log = 1.5) of these compounds resulted in decreased cell viability. However, treating γ-mangostin and α-mangostin in concentration below 10 µM did not affect much with regard to the cell viability (between 80 - 90%). Therefore, a 10 μM concentration of γ-mangostin and α-mangostin was chosen for migration assay to examine their antimigratory effects.

### 4.2. Role of Gamma-Mangostin and Alpha-Mangostin in Migration of MDA-MB-231 Cells

**Figure 2.**
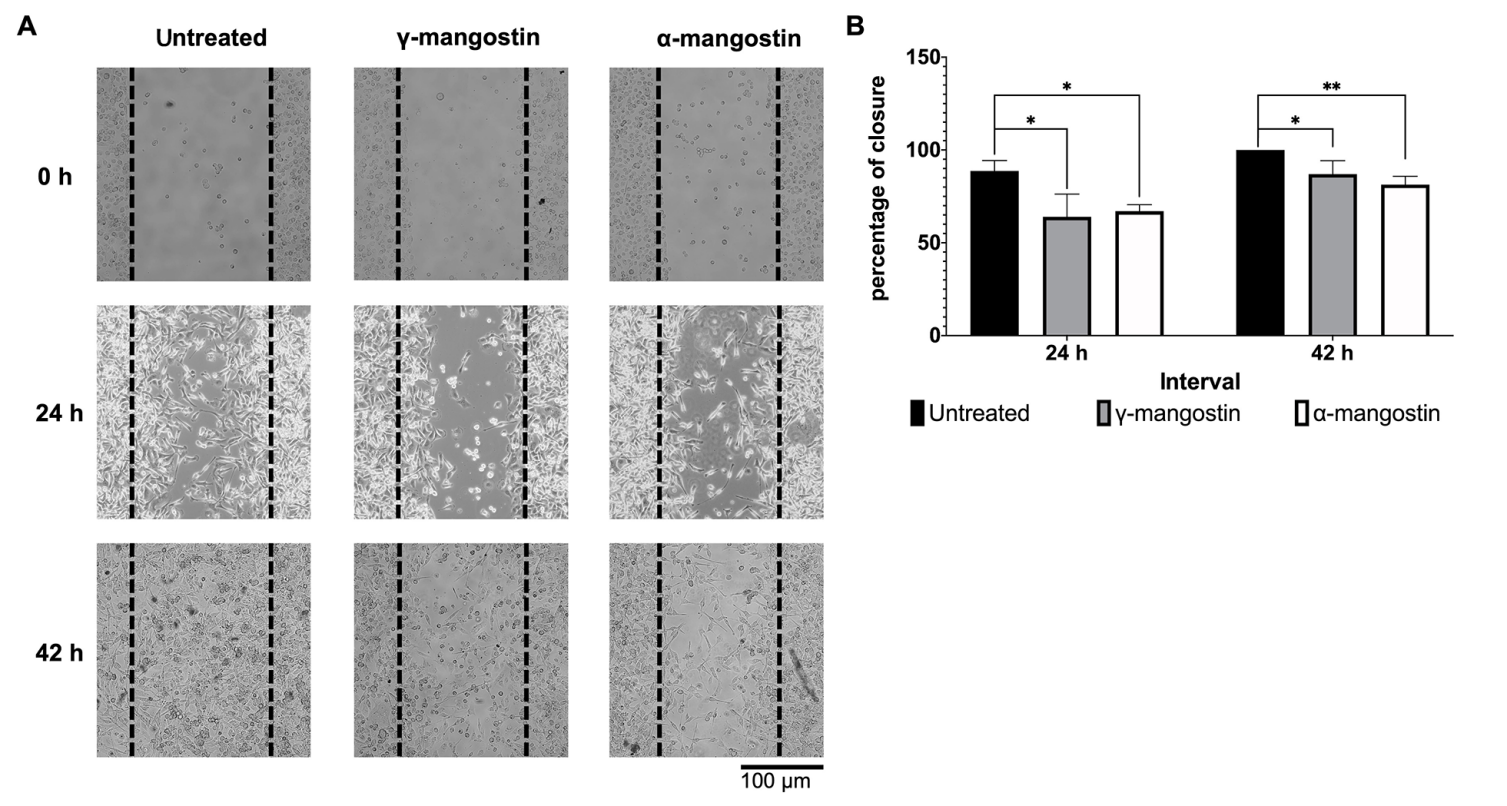
Inhibition of MDA-MB-231 cells migration by gamma-mangostin and alpha-mangostin; A, representative picture of MDA-MB-231 cells after treatment with gamma-mangostin and alpha-mangostin (performing wound healing assay for 24 and 42 hours); B, quantification of cell-free area using imageJ Software and conversion as closure percentage. The data indicate the mean ± standard error (SE); the asterisk represents the significance value (* P < 0.05; ** P < 0.01)

The effect of γ-mangostin and α-mangostin on cell migration was observed by a wound-healing assay. The 10 µM concentration was chosen for further testing, which still allows 80% cell viability (Figure 1A and B). To limit cell proliferation, the starvation medium was applied overnight, and the cells were treated with mitomycin C for 2 hours before the γ-mangostin treatment. It was observed that γ-mangostin effectively inhibited MDA-MB-231 cell migration (P < 0.05); a similar outcome was observed with α-mangostin treatment (Figure 2A). This effect was still evident up to 42 hours of observation (P < 0.05) (Figure 2B). The finding suggests that γ-mangostin and α-mangostin could potentially delay the migration of MDA-MB-231 cells.

### 4.3. Role of Gamma-Mangostin and Alpha-Mangostin in Migration of Regulating Genes of MDA-MB-231 Cells

**Figure 3.**
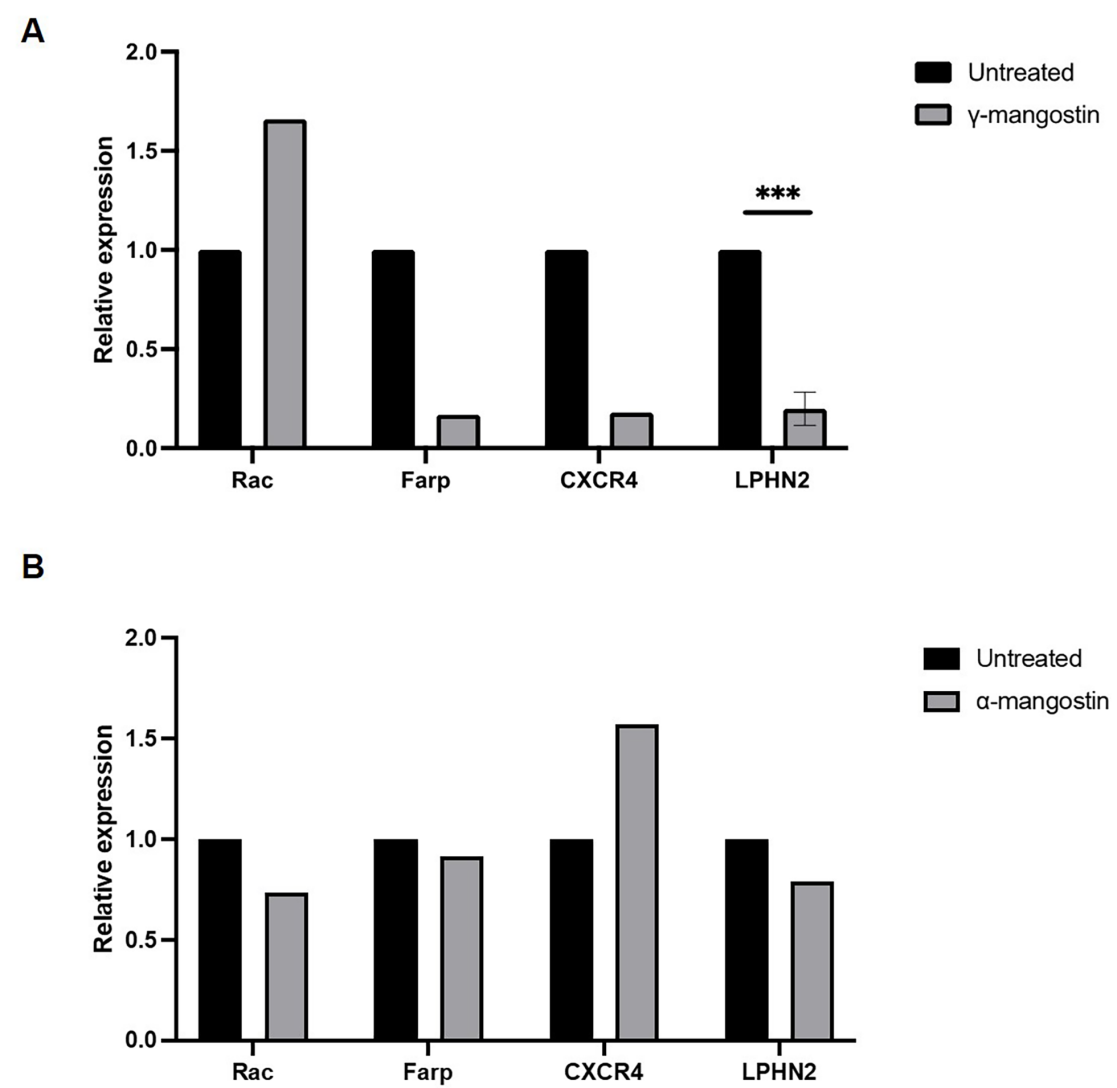
Effect of gamma-mangostin and alpha-mangostin on the transcription level of cell migration-associated genes in MDA-MB-231 Cells. The total RNA was collected from cells that were treated with compound and reverse-transcribed into complementary deoxyribonucleic acid (cDNA) before being amplified with Rac, *Farp*, CXCR4, and *LPHN2* primers using SYBR-probed real-time PCR. The relative expression from A, γ-mangostin and B, α-mangostin group after normalized with the *GAPDH* gene (*** P < 0.001 from three replicates)

Various proteins are involved in the regulation of cell migration. To uncover the mechanism by which γ-mangostin and α-mangostin act as anti-migratory agents, the present study investigated the gene expression of *Rac1*, *Farp*, CXCR4, and *LPHN2* using RT-qPCR. Treatment of γ-mangostin downregulated the expression of *Farp*, CXCR4, and *LPHN2* (Figure 3A). However, α-mangostin treatment did not alter the expression of these genes (Figure 3B). Although this experiment was only performed in one measurement, it was expected to provide valuable information regarding the effect of the substances on the expression of migration marker genes. Nevertheless, this phenomenon suggests that the unique migration inhibitory effect of γ-mangostin correlated to suppressing these gene expressions, which might not be the case for α-mangostin molecular activities in MDA-MB-231 cell migration.

### 4.4. Role of Gamma-Mangostin and Alpha-Mangostin in MDA-MB-231 Cellular ROS Production

**Figure 4.**
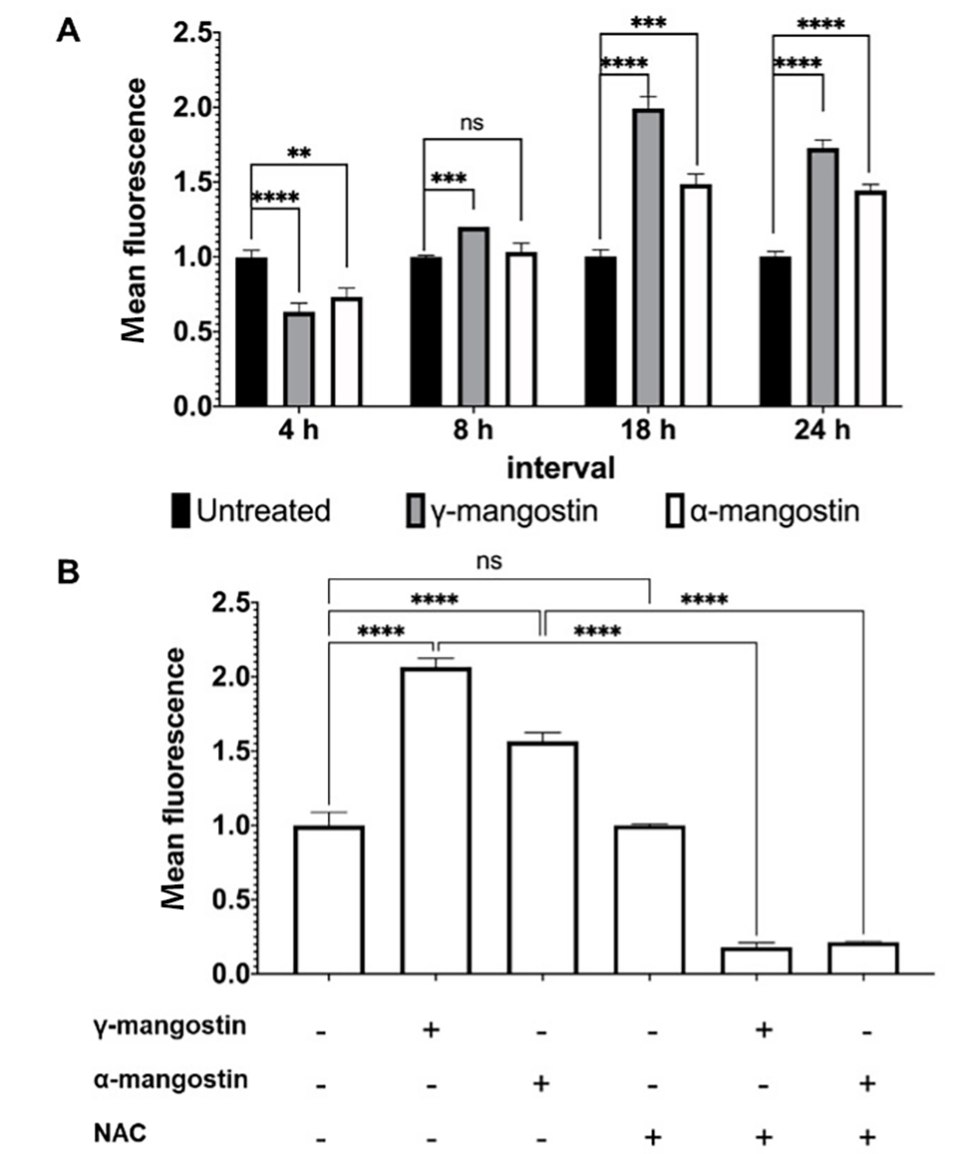
Induction of reactive oxygen species (ROS) production in MDA-MB-231 cells by gamma-mangostin and Alpha-Mangostin. A, cells were treated with 10 µM γ-mangostin or 10 µM α-mangostin for 24 hours. The reactive oxygen species (ROS) amount was measured according to the intensity of 2,7-dichlorofluorescein (DCF) by flow cytometry. B, cells were exposed with or without 5 mM NAC for 1 hour before being incubated with 10 µM γ-mangostin or 10 μM α-mangostin for 24 hours. The detection of cellular ROS levels was determined and quantified by flow cytometry. The data were shown as the average of 3 data ± standard deviation (SD); nevertheless, the asterisk represented the significance value (**P < 0.01; *** P < 0.001; **** P < 0.0001)

Intracellular ROS is essential in the growth and invasiveness of cancer cells; therefore, its level is higher in cancer cells than in normal cells (32). If ROS levels exceed the capacity of tumor cells to metabolize, they can inhibit proliferation and migration, leading to cancer cell death (33). The present study treated cells with γ-mangostin or α-mangostin and monitored ROS levels across 24 hours at various intervals (4, 8, 18, and 24 hours) since cellular ROS have a short lifespan (34). In the current study, it was observed that ROS accumulation decreased after 4 hours of γ-mangostin or α-mangostin treatment and started to rise (P < 0.0001) after a longer incubation period of up to 24 hours. A similar result was also demonstrated in α-mangostin-treated cells (Figure 4A). The addition of the ROS scavenger NAC prior to the treatment reduced the amount of ROS induced by γ-mangostin and α-mangostin (P < 0.0001) (Figure 4B). Overall, it appeared that γ-mangostin and α-mangostin changed ROS levels in MDA-MB-231 cells, suggesting that γ-mangostin and α-mangostin regulate cell activities, including viability and migration, by modulating ROS levels.

### 4.5. Molecular Docking Analysis of Gamma-Mangostin and Alpha-Mangostin to the CXCR4 Receptor

Beyond assessing messenger RNA (mRNA) level expression, the insilico method was also conducted through a molecular docking study to assess the potential activity of γ-mangostin and α-mangostin on CXCR4. Among the gene targets, only *CXCR4 *was found in the PDB database (PDB ID: 3ODU). The *CXCR4 *gene is one of the GPCRs and, together with its ligand CXCL12, plays a significant role in cell migration (35).

**Figure 5.**
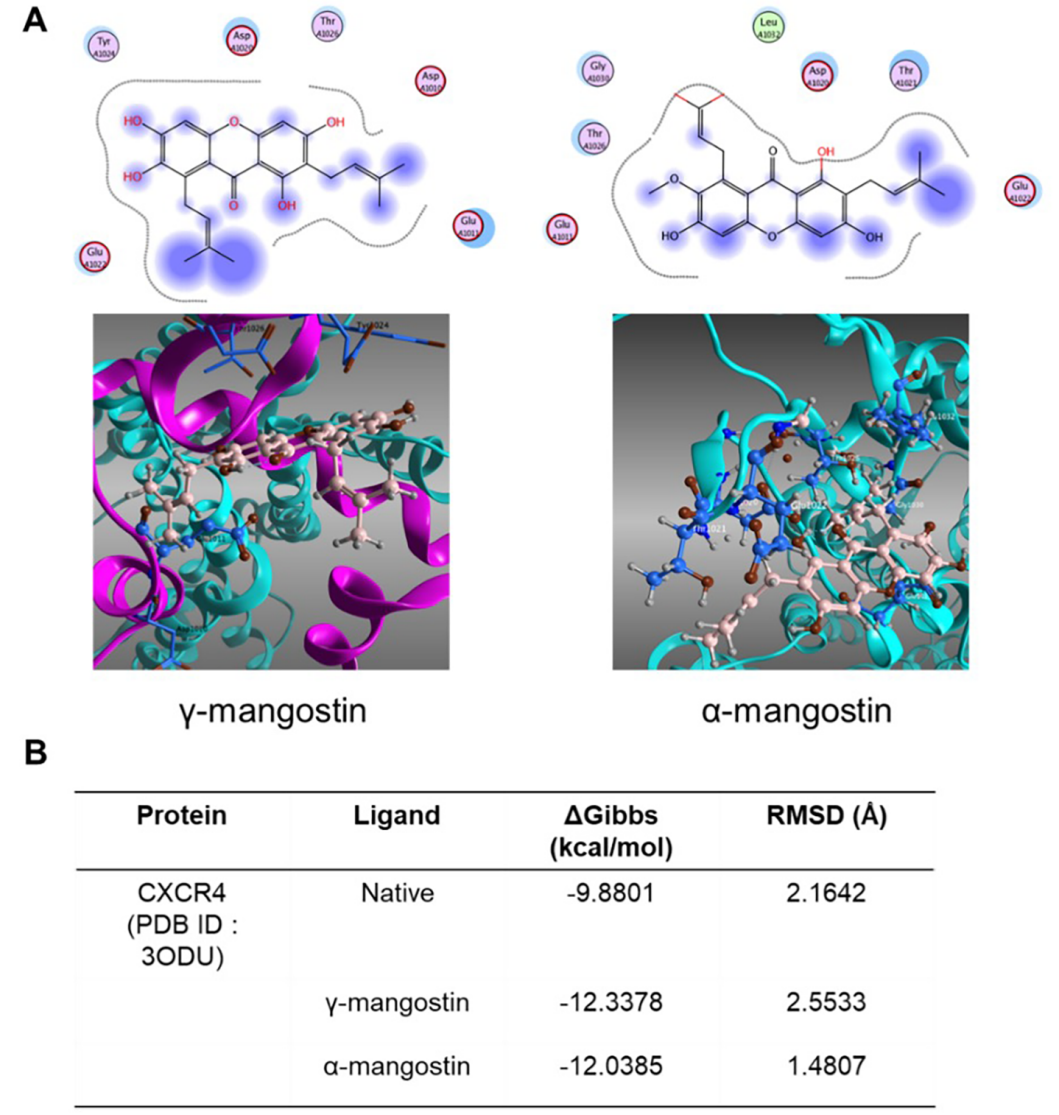
Molecular docking simulation of gamma-mangostin and alpha-mangostin in *CXCR4 *Receptor; A, two-dimensional (2D) and three-dimensional (3D) visualization of the Ligand gamma-mangostin (Left) and alpha-mangostin (Right) in *CXCR4 *Receptor (PDB ID: 3ODU) Generated from Molecular Operating Environment (MOE) Software (Version 2010.12); B, affinity binding and root mean square deviation (RMSD) value of gamma-mangostin and alpha-mangostin (and IT1t, a small molecule antagonist) after docking simulation

Docking simulation predicted that the purple area in γ-mangostin and α-mangostin was in proximity to the *CXCR4 *amino acid, although these compounds did not directly bind to CXCR4. The functional group of γ-mangostin was surrounded by six amino acid residues; however, α-mangostin was surrounded by seven amino acid residues (Figure 5A). Further analysis revealed that γ-mangostin and α-mangostin had lower energy scores than the IT1t, a small molecule antagonist. These interactions suggest these compounds have a stronger binding affinity to the receptor than IT1t (Figure 5B). 

## 5. Discussion

To better understand how γ-mangostin and α-mangostin inhibit MDA-MB-231 breast cancer cell migration, the present study examined the expression of several genes associated with cancer cell migration. The obtained findings suggest that γ-mangostin is capable of downregulating the transcription level of CXCR4, *Farp*, and *LPHN2*, unlike α-mangostin, which did not impact the expression of these genes. This effect suggests the presence of a specific mechanism through which γ-mangostin targets these genes, warranting further research to identify potential drug targets associated with γ-mangostin.

Notably, CXCR4, a chemokine receptor for the ligand CXCL12, is known for its significant role in cell proliferation, adhesion, and migration, which is also intricately associated with invasion and metastasis through Ras/Raf signaling (16, 36). Meanwhile, *Farp*, implicated in F-actin polymerization (37), has been demonstrated to be involved in cancer (38). Although α-mangostin treatment did not alter the transcription level of those genes, a study by Nalla et al. (39) reported that this compound inhibited MMP-2 protein and triggered E-cadherin, partly inhibiting MDA-MB-231 cell migration and invasion. Therefore, it is likely that there are different molecular mechanisms between γ-mangostin and α-mangostin to inhibit cancer cell migration. Since the data are limited, these phenomena should be clarified further with more comprehensive analyses, including time points experiments and protein level expressions.

Beyond assessing mRNA level expression, in silico methods were also used to evaluate the potential activity of γ-mangostin on CXCR4. The insilico experiment is the preliminary study of the two compounds regarding their affinities to interact with *CXCR4 *as the main target protein. The results revealed that the binding energy of γ-mangostin and α-mangostin was lower than the comparator antagonist, suggesting substantial potential for γ-mangostin and α-mangostin to inhibit the protein’s activity. The promising *CXCR4 *antagonist candidate, BPRCX807, is known for binding into primary critical residues in the major subpocket, thereby exhibiting a remarkable effect to inhibit CXCR4-CXCL12 interaction (19). Therefore, the evaluation of the molecular dynamics of γ-mangostin and α-mangostin in CXCR4-CXCL12 binding should be further elucidated.

Reactive oxygen species are by-products of cellular metabolism and participate in various signal transduction responsible for cancer cell proliferation and invasion (32). Tumor cells naturally have higher basal ROS levels than normal cells due to disrupted redox homeostasis in cancer cells (40), and it is known that TNBC cells exhibit the highest ROS among other subtypes of breast cancer (10). However, the overproduction of endogenous ROS, augmented by exogenous chemotherapy agents, can induce oxidative damage to the cancer cells as the redox level becomes unbalanced, leading to cancer cell death.

In the present study, γ-mangostin and α-mangostin increased ROS levels, and NAC addition nearly eliminated the ROS levels induced by these compounds. Other studies have reported a similar effect of α-mangostin on inducing cellular ROS production (41, 42). Alpha-mangostin inhibited catalase activity in TNBC 4T1 cells but not antioxidant activity, resulting in the pro-oxidant effect on cancer cells (42). Moreover, α-mangostin-induced ROS overproduction might activate the PI3K/AKT signaling pathway, inhibiting cancer cell proliferation, migration, and induction of mitochondrial dysfunction that mediates apoptosis in MDA-MB-231 cells (23). Another finding also revealed that α-mangostin enhanced higher ROS accumulation in hepatoblastoma HepG2 cells than in hepatocyte WRL-68 cells, which could imply the selectivity of α-mangostin in cells (43).

Although there are numerous reports of α-mangostin’s activity in increasing ROS levels in cancer cells, similar activity by γ-mangostin in human cancer cells is less documented. The treatment of γ-mangostin in human colorectal cancer cell line HT29 showed enhanced intracellular ROS levels and intervened mitochondria function, causing cancer cell death (25). Previous studies have focused on γ-mangostin’s protective effect against oxidative damage in neurons and ischemia-induced myocardial cell injury (44, 45). Therefore, the current study provides further evidence for γ-mangostin’s involvement in ROS accumulation in cancer cell death, indicating the necessity for more in-depth investigation.

Numerous researchers have reported ROS involvement in cancer cell migration that is activated through several pathways (46). The ROS formation affects the enzymatic activity of gelatinases MMPs (i.e., MMP-2 and MMP-9) that are controlled through MAPK signaling (47); it also enhances *CXCR4 *transcription that is mediated by HIF1α (48). On the other hand, the excessive oxidative stress in cancer cells causes the imbalance of cell homeostasis and activates the apoptosis pathway (49), which later becomes one of the strategic approaches for cancer therapy. The current study provides novel knowledge that revealed the γ-mangostin effect on suppressing *CXCR4 *transcription, thereby inhibiting cancer cell migration and cell proliferation. The present study’s findings stipulate further exploration to comprehend how γ-mangostin acts as a potential anticancer candidate that targets multiple pathways associated with cancer cell survival and migration. At least, the data here supported the potency of γ-mangostin development for therapy in metastatic breast cancer.

### 5.1. Conclusions

In conclusion, the present study demonstrated that γ-mangostin exhibits antimigratory action on MDA-MB-231 breast cancer cells, mainly impacting the downregulation of CXCR4, *Farp*, and *LPHN2* genes, but not the case with α-mangostin. Molecular docking simulations further suggested the potential for γ-mangostin to inhibit CXCR4. Likewise, the obtained findings suggest that the effect of elevated ROS levels under γ-mangostin and α-mangostin treatment can also be associated with inhibiting MDA-MB-231 cell proliferation and migration with regard to *CXCR4 *activity inhibition.

## Data Availability

The dataset presented in the study is available on request from the corresponding author during submission or after publication. The data are not publicly available due to the privacy.

## References

[1] Hamurcu Z, Delibasi N, Gecene S, Sener EF, Donmez-Altuntas H, Ozkul Y (2018). Targeting LC3 and Beclin-1 autophagy genes suppresses proliferation, survival, migration and invasion by inhibition of Cyclin-D1 and uPAR/Integrin beta1/ Src signaling in triple negative breast cancer cells.. J Cancer Res Clin Oncol..

[2] Al-Mahmood S, Sapiezynski J, Garbuzenko OB, Minko T (2018). Metastatic and triple-negative breast cancer: Challenges and treatment options.. Drug Deliv Transl Res..

[3] Yadav AR, Mohite SK (2020). Cancer-A silent killer: An overview.. Asian J Pharmaceutical Res..

[4] Patidar A, Shivhare SC, Ateneriya U, Choudhary S (2012). A Comprehensive Review on Breast Cancer.. Asian J Nursing Edu Res..

[5] Damaskos C, Garmpi A, Nikolettos K, Vavourakis M, Diamantis E, Patsouras A (2019). Triple-negative breast cancer: The progress of targeted therapies and future tendencies.. Anticancer Res..

[6] Eldhose E, Gowramma B, Mohammed M, Kalirajan R, Kaviarasan L (2019). Translational Chemotherapy for triple negative Breast Cancer-A Review on significance of poly (ADP-ribose) polymerase 1 (PARP 1) inhibitors.. Res J Pharmacy Technol..

[7] Abu Samaan TM, Samec M, Liskova A, Kubatka P, Busselberg D (2019). Paclitaxel's mechanistic and clinical effects on breast cancer.. Biomolecules..

[8] Wahba HA, El-Hadaad HA (2015). Current approaches in treatment of triple-negative breast cancer.. Cancer Biol Med..

[9] Spathas N, Economopoulou P, Cheila M, Kotsantis I, Fanouriakis A, Kassara D (2018). Inflammatory arthritis induced by pembrolizumab in a patient with head and neck squamous cell carcinoma.. Front Oncol..

[10] Sarmiento-Salinas FL, Delgado-Magallon A, Montes-Alvarado JB, Ramirez-Ramirez D, Flores-Alonso JC, Cortes-Hernandez P (2019). Breast cancer subtypes present a differential production of reactive oxygen species (ros) and susceptibility to antioxidant treatment.. Front Oncol..

[11] Malla R, Surepalli N, Farran B, Malhotra SV, Nagaraju GP (2021). Reactive oxygen species (ROS): Critical roles in breast tumor microenvironment.. Crit Rev Oncol Hematol..

[12] Redza-Dutordoir M, Averill-Bates DA (2016). Activation of apoptosis signalling pathways by reactive oxygen species.. Biochim Biophys Acta..

[13] Gupta N, Mohan CD, Shanmugam MK, Jung YY, Chinnathambi A, Alharbi SA (2023). CXCR4 expression is elevated in TNBC patient derived samples and Z-guggulsterone abrogates tumor progression by targeting CXCL12/CXCR4 signaling axis in preclinical breast cancer model.. Environ Res..

[14] Xu C, Zhao H, Chen H, Yao Q (2015). CXCR4 in breast cancer: Oncogenic role and therapeutic targeting.. Drug Des Devel Ther..

[15] Koch C, Fischer NC, Puchert M, Engele J (2022). Interactions of the chemokines CXCL11 and CXCL12 in human tumor cells.. BMC Cancer..

[16] Guo F, Wang Y, Liu J, Mok SC, Xue F, Zhang W (2016). CXCL12/CXCR4: A symbiotic bridge linking cancer cells and their stromal neighbors in oncogenic communication networks.. Oncogene..

[17] Chetram MA, Hinton CV (2013). ROS-mediated regulation of CXCR4 in cancer.. Front Biol (Beijing)..

[18] De Clercq E (2019). Mozobil(R) (Plerixafor, AMD3100), 10 years after its approval by the US Food and Drug Administration.. Antivir Chem Chemother..

[19] Song JS, Chang CC, Wu CH, Dinh TK, Jan JJ, Huang KW (2021). A highly selective and potent CXCR4 antagonist for hepatocellular carcinoma treatment.. Proc Natl Acad Sci U S A..

[20] Kalick LS, Khan HA, Maung E, Baez Y, Atkinson AN, Wallace CE (2023). Mangosteen for malignancy prevention and intervention: Current evidence, molecular mechanisms, and future perspectives.. Pharmacol Res..

[21] Setyawati LU, Nurhidayah W, Khairul Ikram NK, Mohd Fuad WE, Muchtaridi M (2023). General toxicity studies of alpha mangostin from Garcinia mangostana: A systematic review.. Heliyon..

[22] Lee Y, Ko K, Shi M, Liao Y, Chiang T, Wu P (2010). α‐Mangostin, A Novel Dietary Xanthone, Suppresses TPA‐Mediated MMP‐2 and MMP‐9 Expressions through the ERK Signaling Pathway in MCF‐7 Human Breast Adenocarcinoma Cells.. J Food Sci..

[23] Zhu X, Li J, Ning H, Yuan Z, Zhong Y, Wu S (2021). alpha-mangostin induces apoptosis and inhibits metastasis of breast cancer cells via regulating RXRalpha-AKT signaling pathway.. Front Pharmacol..

[24] Herdiana Y, Wathoni N, Shamsuddin S, Muchtaridi M (2021). alpha-mangostin nanoparticles cytotoxicity and cell death modalities in breast cancer cell lines.. Molecules..

[25] Chang HF, Yang LL (2012). Gamma-mangostin, a micronutrient of mangosteen fruit, induces apoptosis in human colon cancer cells.. Molecules..

[26] Novitasari D, Kato J, Ikawati M, Putri DDP, Wulandari F, Widyarini S (2023). PGV-1 permanently arrests HepG2 cells in M phase and inhibits liver carcinogenesis in DMH-induced rats.. J Applied Pharmaceutical Sci..

[27] Novitasari D, Meiyanto E, Kato J, Jenie RI (2022). Antimigratory Evaluation from Curcumin-Derived Synthetic Compounds PGV-1 and CCA-1.1 on HCC1954 and MDA-MB-231 Cells.. Indonesian J Cancer Chemoprevention..

[28] Li X, Li P, Chang Y, Xu Q, Wu Z, Ma Q (2014). The SDF-1/CXCR4 axis induces epithelial-mesenchymal transition in hepatocellular carcinoma.. Mol Cell Biochem..

[29] Novitasari D, Jenie RI, Kato JY, Meiyanto E (2023). Chemoprevention curcumin analog 1.1 promotes metaphase arrest and enhances intracellular reactive oxygen species levels on TNBC MDA-MB-231 and HER2-positive HCC1954 cells.. Res Pharm Sci..

[30] Lestari B, Nakamae I, Yoneda-Kato N, Morimoto T, Kanaya S, Yokoyama T (2019). Pentagamavunon-1 (PGV-1) inhibits ROS metabolic enzymes and suppresses tumor cell growth by inducing M phase (prometaphase) arrest and cell senescence.. Sci Rep..

[31] Meiyanto E, Novitasari D, Utomo RY, Susidarti RA, Putri DDP, Kato J (2022). Bioinformatic and molecular interaction studies Uncover That CCA-1.1 AND PGV-1 differentially target mitotic regulatory protein and have a synergistic effect against leukemia cells.. Indonesian J Pharmacy..

[32] De Sa Junior PL, Camara DAD, Porcacchia AS, Fonseca PMM, Jorge SD, Araldi RP (2017). The roles of ROS in cancer heterogeneity and therapy.. Oxid Med Cell Longev..

[33] Hayes JD, Dinkova-Kostova AT, Tew KD (2020). Oxidative stress in cancer.. Cancer Cell..

[34] Murphy MP, Bayir H, Belousov V, Chang CJ, Davies KJA, Davies MJ (2022). Guidelines for measuring reactive oxygen species and oxidative damage in cells and in vivo.. Nat Metab..

[35] Sun Y, Mao X, Fan C, Liu C, Guo A, Guan S (2014). CXCL12-CXCR4 axis promotes the natural selection of breast cancer cell metastasis.. Tumour Biol..

[36] Shi Y, Riese D2, Shen J (2020). The Role of the CXCL12/CXCR4/CXCR7 Chemokine Axis in Cancer.. Front Pharmacol..

[37] Cheadle L, Biederer T (2012). The novel synaptogenic protein Farp1 links postsynaptic cytoskeletal dynamics and transsynaptic organization.. J Cell Biol..

[38] Giampieri S, Manning C, Hooper S, Jones L, Hill CS, Sahai E (2009). Localized and reversible TGFbeta signalling switches breast cancer cells from cohesive to single cell motility.. Nat Cell Biol..

[39] Nalla LV, Dharavath A, Behera SK, Khairnar A (2023). Alpha mangostin inhibits proliferation, migration, and invasion of human breast cancer cells via STAT3 inhibition.. Advances in Cancer Biology - Metastasis..

[40] Saikolappan S, Kumar B, Shishodia G, Koul S, Koul HK (2019). Reactive oxygen species and cancer: A complex interaction.. Cancer Lett..

[41] Sheng X, Li J, Zhang C, Zhao L, Guo L, Xu T (2019). alpha-Mangostin promotes apoptosis of human rheumatoid arthritis fibroblast-like synoviocytes by reactive oxygen species-dependent activation of ERK1/2 mitogen-activated protein kinase.. J Cell Biochem..

[42] Cruz-Gregorio A, Aranda-Rivera AK, Aparicio-Trejo OE, Medina-Campos ON, Sciutto E, Fragoso G (2023). alpha-Mangostin induces oxidative damage, mitochondrial dysfunction, and apoptosis in a triple-negative breast cancer model.. Phytother Res..

[43] Harliansyah H, Rahmah NA, Kuslestari K (2021). α-Mangosteen as An Oxidative Inhibitor in Hepatocellular Carcinoma.. Indonesian J Cancer Chemoprevention..

[44] Baek JY, Jung K, Kim YM, Kim HY, Kang KS, Chin YW (2021). Protective Effect of gamma-mangostin Isolated from the Peel of Garcinia mangostana against Glutamate-Induced Cytotoxicity in HT22 Hippocampal Neuronal Cells.. Biomolecules..

[45] Dong X, Zhu J (2023). γ-Mangostin alleviates myocardial ischemia-reperfusion injury by up-regulating SIRT3.. Tropical J Pharmaceutical Res..

[46] Tochhawng L, Deng S, Pervaiz S, Yap CT (2013). Redox regulation of cancer cell migration and invasion.. Mitochondrion..

[47] Saari H, Sorsa T, Lindy O, Suomalainen K, Halinen S, Konttinen YT (1992). Reactive oxygen species as regulators of human neutrophil and fibroblast interstitial collagenases.. International J tissue reactions..

[48] Li S, Deng Y, Feng J, Ye W (2009). Oxidative preconditioning promotes bone marrow mesenchymal stem cells migration and prevents apoptosis.. Cell Biol Int..

[49] Perillo B, Di Donato M, Pezone A, Di Zazzo E, Giovannelli P, Galasso G (2020). ROS in cancer therapy: The bright side of the moon.. Exp Mol Med..

